# Interbirth interval and history of previous preeclampsia: a case–control study among multiparous women

**DOI:** 10.1186/1471-2393-13-244

**Published:** 2013-12-27

**Authors:** Arusyak Harutyunyan, Haroutune Armenian, Varduhi Petrosyan

**Affiliations:** 1 American University of Armenia, College of Health Sciences, Baghramyan 40, Yerevan, Armenia; 2 Department of Epidemiology, University of California, Fielding School of Public Health, Los Angeles, USA

## Abstract

**Background:**

Preeclampsia is a disorder with a reported incidence of 2%-8% among all pregnancies, accounting for more than 50,000 deaths worldwide each year. In low- and middle- income countries maternal/perinatal morbidity and mortality associated with preeclampsia are high due to the lack of proper prenatal and hospital care and limited access to neonatal intensive care. The objectives of our study were to determine the association of long interbirth interval (IBI) and preeclampsia and to investigate the interactions between long IBI and other risk factors among multiparous women in Yerevan, Armenia.

**Methods:**

We conducted a hospital-based case–control study among 36 multiparous women with preeclampsia (cases) and 148 without preeclampsia (controls) during their last pregnancy, selected from the two largest maternity hospitals in Armenia. The data were collected through telephone-based structured interviews and analyzed using STATA software. The study applied univariate and multivariate logistic regression analyses.

**Results:**

The study found a significant interaction between IBI and previous history of preeclampsia. Among women without a history of previous preeclampsia, the odds of having preeclampsia among women with long IBI (greater than or equal to five years) was 6.88 time higher compared to those with short IBI (CI: 1.75-27.05; p = 0.006) after adjusting for confounders; among women with a history of previous preeclampsia the odds ratio was 0.60 (CI: 0.07-4.99; p = 0.638). The final fitted model for preeclampsia among multiparous women who had planned their pregnancies included IBI, time to pregnancy, Body Mass Index, method of contraception and household monthly income.

**Conclusions:**

Long IBI appeared to be a strong risk factor for preeclampsia development only among women without a history of previous preeclampsia. This finding may contribute to a new approach in understanding the etiology of preeclampsia and may be useful for developing further recommendations for this particular subgroup of women that are at higher risk for preeclampsia development in subsequent pregnancies.

## Background

Preeclampsia has been termed as a “disease of theories”, reflecting the confusion that surrounds its causes and pathophysiology [1]. Preeclampsia is a disorder with a reported incidence of 2%-8% among all pregnancies, accounting for more than 50,000 deaths worldwide each year [2]. Even in countries with low maternal mortality rates, a substantial proportion of maternal mortalities is due to preeclampsia/eclampsia [3]. Preeclampsia also affects the infants’ well-being, leading to poor intrauterine growth, prematurity and high perinatal mortality rates [4]. In developing countries, the lack of proper prenatal and hospital care, and limited access to neonatal intensive care lead to higher maternal/perinatal morbidity and mortality associated with preeclampsia [2-5]. Maternal mortality in Armenia has declined from 34.7 deaths per 100,000 live births in 1995 to 13.2 deaths per 100,000 live births in 2011, but it still exceeds the average rates for European Union (EU) new member states (less than 10 maternal deaths per 100,000 live births) and is noticeably higher than in EU-15 countries (less than 6 maternal deaths per 100,000 live births) [6,7]. Preeclampsia is among one of the three leading causes of maternal mortality worldwide, including Armenia [2].

Despite extensive research exploring the risk factors and the management of preeclampsia, there has been no improvements in predicting who would develop preeclampsia, and there are no protocols for prevention or treatment for this condition other than delivery (even if it is a preterm delivery).

Preeclampsia in primiparous women is 4–5 times more prevalent than multiparous women who have had a previous normal pregnancy [8-11]. Moreover, multiparous women have milder symptoms and most are recurrent cases [12]. Other factors associated with preeclampsia are maternal age [8,10], family history of preeclampsia [8,13], level of education [14], high Body Mass Index (BMI) [8,9,15,16], pre-existing medical conditions such as chronic hypertension, renal diseases, diabetes- including gestational diabetes [8,15], and others.

Few studies have investigated risk factors for preeclampsia development among multiparous women. Reported risk factors of preeclampsia for multiparous women include interbirth interval (IBI) [15,17-19], partner change [11,20,21], previous low birth weight delivery and preterm deliveries [18], and history of previous preeclampsia [8-11,15]. The literature suggests that longer IBI increases the risk of preeclampsia, indicating that the protective effect of past pregnancies may decline over time or that other time dependent factors contribute to increased risk [22]. Although different studies have used different time intervals, most report a significant association between long IBI and increased risk of preeclampsia. Although the exact length of the interval where the risk of preeclampsia begins to increase is not clear, IBIs of five years or more are associated with increased risk of preeclampsia [17].

The objectives of this study were to explore risk factors (including IBI) for preeclampsia and to investigate interactions between IBI and other risk factors among reproductive age multiparous women in Yerevan, Armenia.

## Methods

We conducted a case–control study among women of reproductive age (18–44) living in Yerevan who were admitted to the Institute of Obstetrics (Perinatology), Gynecology and Reproductive Health and the Erebuni Medical Center for delivery from the 1^st^ of January 2008 to the 1^st^ of April 2009. These two maternity hospitals are the largest referral tertiary care centers in Armenia and are responsible for one-third of all deliveries in Yerevan.

Cases were women living in Yerevan who were diagnosed with preeclampsia in the last pregnancy in the selected maternity hospitals. The definition of a preeclampsia case status was based on the US National Heart Lung and Blood Institute (NHLBI) criteria for diagnosis of this condition [23]. Controls were defined as reproductive age women living in Yerevan that gave birth in the same maternity hospitals with no diagnosis of preeclampsia during their last pregnancy.

After receiving permission from the directors of the two maternity hospitals, a member of the research team, an OB/GYN, identified cases and controls while completing data abstraction forms during the review of the medical records. All cases who were identified were selected for inclusion into the study. The study used an incidence-density sampling approach for selecting controls. Three controls without preeclampsia were randomly sampled using a random number table from all eligible women that gave birth in the selected maternity hospitals in the same month when a case was diagnosed with preeclampsia. Overall, 102 eligible cases and 306 controls from the registries of the two maternity hospitals were selected for a larger study. From this larger study population, the current study excluded all primiparous women and conducted an analysis of the remaining 36 cases and 148 controls.

An interviewer-administered structured questionnaire was used for data collection by telephone for both cases and controls Additional file 1. During the telephone interview, oral consent was obtained from each participant. The research team designed the questionnaire, which included questions adopted from questionnaires used in other studies to investigate risk factors for preeclampsia development and questions added by the researchers adopted from other instruments [24-26]. The questionnaire was pre-tested among five reproductive age women through telephone interviews.

The dependent (outcome) variable of the study was preeclampsia status during the last pregnancy that was clinically confirmed by a doctor at the maternity hospital and checked by the researchers against the US NHLBI criteria. The main independent variables were IBI, age, educational level, parity, type of contraceptive used within one year before the pregnancy, time to pregnancy (TTP), family history of preeclampsia, history of previous preeclampsia, BMI, chronic hypertension, and renal disease.

The IBI was calculated as the interval in years between the dates of the two last deliveries. The IBI was analyzed both as a continuous and a dichotomous variable defined as short (<5 years) and long (≥5 years). The TTP, as a marker of fecundity, was defined as the time interval in months required for a couple to conceive from the start of unprotected intercourse and was used as dichotomous variable with a cut-point of 12 months. The method of contraception was included as a dichotomous variable defined as barrier (condoms, diaphragms, spermicides and withdrawal) and non-barrier methods of contraception [27]. The BMI, calculated as pre-pregnancy weight (kilograms) per height squared (meters squared), and age were analyzed as continuous variables.

To assess the relationships between each independent variable and the dependent variable and to identify confounders for the relationship between IBI and preeclampsia status, the study performed univariate logistic regressions. All identified confounders were included in the multivariate logistic regression model. Categorical data were converted into “dummy” variables for the regression analysis. The study applied multivariate logistic regression models to control for potential confounders and explore potential effect modification and, ultimately, to calculate the adjusted odds ratios and 95% confidence intervals to estimate the strength of associations between independent and dependent variables. Multivariate logistic regression analysis was also used to produce a final fitted model. Each full model was tested against the nested model using Akaike’s Information Criteria (AIC) which is an approximation to the cross-validated prediction error (e.g. criteria for determining the “best model”). The model fit was tested with Hosmer-Lemeshow goodness of fit test. Using the alpha error of 0.05, we calculated the actual statistical power based on the proportions of the primary variable (IBI) and the actual sample size of 36 cases and 148 controls and the power was 0.99.

The Institutional Review Board/Committee on Human Research (IRB) of the American University of Armenia approved the study.

## Results

From 102 eligible cases and 306 controls selected from the registries of two maternity hospitals for telephone-based interviews the study team failed to contact 23 subjects due to different reasons. Out of the contacted 385 subjects 17 refused to participate (4 cases and 13 controls) and 89 cases and 279 controls completed the interviews. The response rate was 95.7% for cases and 95.5% for controls. We analysed the data of 184 multiparous women subsampled from the original sample. Table 1 presents descriptive statistics of the 36 multiparous cases and 148 controls. Cases were older compared to controls, with the mean age of cases equal to 31.3 (SD: 5.9) vs. 28.1 (SD: 4.3). The cases had a higher BMI than controls, with 25.5 kg/m^2^ (SD: 4.0) vs. 21.6 kg/m^2^ (SD: 3.6). Cases were more likely to have an IBI more than five years compared to controls, with 63.9% vs. 26.7% respectively. Cases and controls were statistically significantly different in age, BMI, IBI, renal disease, TTP, family history of preeclampsia, number of stillbirths and history of previous preeclampsia.

**Table 1:** Descriptive characteristics of multiparous preeclampsia cases and their multiparous controls

**Variable**	**Cases n = 36**	**Controls n = 148**	***P *** **value**
Age at delivery (years) (SD)*	31.3 (5.9)	28.1 (4,3)	<0.0005
BMI (kg/m^2^) (SD)*	25.5 (4.0)	21.6 (3.6)	<0.0005
Renal disease, % (n)	13.9 (5)	2.7 (4)	0.015
Women’s positive Rh factor, % (n)	76.5 (26)	91.2 (135)	0.016
Household monthly expenditure >100,000 AMD, % (n)	55.9 (19)	60.2 (77)	0.065
Barrier method of contraception, % (n)	47.2 (17)	34.5 (51)	0.155
Time to pregnancy ≥12 months, % (n)	40.0 (8)	11.0 (10)	0.004
Family history of preeclampsia, % (n)	20.6 (7)	5.1 (7)	0.008
IBI (years) (SD)*	7.3 (4.5)	4.2 (2.8)	<0.0005
Long IBI (≥5years), % (n)	63.9 (23)	26.7 (39)	0.0005
Stillbirth in previous pregnancies, % (n)	13.9 (5)	4.1 (6)	0.026
History of previous preeclampsia, % (n)	47.2 (17)	4.1 (6)	<0.0005

BMI, Body Mass Index; IBI, Interbirth Interval.

*Mean (standard deviation).

Univariate logistic regression analysis showed that age at delivery, BMI, renal disease and TTP were significantly associated with both preeclampsia status and IBI, therefore confounding the relationship between preeclampsia status and IBI.

Table 2 presents the association between preeclampsia status and independent variables with no adjustment and with adjustment for confounding variables. After adjusting for age, BMI, and renal disease, the odds of having preeclampsia was higher among women with long IBI compared to women with short IBI (OR = 2.90; 95% CI: 1.07-7.86; p = 0.036). Although TTP was identified as a confounder, IBI was not adjusted for it because of a large number of missing values for this variable as only those who planned their pregnancy reported TTP (111 multiparous women).

**Table 2:** Association between interbirth interval and preeclampsia status with and without adjustment for confounders

**Variable**	**Univariate analysis**		**Multivariate analysis**	
	**Unadjusted OR**	**95% CI**	Adjusted OR*	**95% CI**
IBI (years)				
<5	1.00			
≥5	5.26	2.39-11.57	2.90	1.07-7.86
Age at delivery (years)	1.14	1.06-1.24	1.03	0.93-1.14
BMI (kg/m2)	1.26	1.14-1.40	1.23	1.11-1.37
Renal disease				
No	1.00		1.00	
Yes	5.81	1.47-22.87	0.98	0.16-5.96

BMI, Body Mass Index; IBI, Interbirth Interval.

*Adjusted for all variables in Table 2.

### IBI as a dichotomous variable

The research team checked for possible interactions between IBI and other independent variables and identified a statistically significant interaction between history of previous preeclampsia and the IBI (Table 3). The interaction term between IBI with the cut-point of 5 years and history of previous preeclampsia after adjusting for age, BMI and renal disease was 0.09 (95% CI: 0.01-0.96; p = 0.046). Among women without history of previous preeclampsia after adjusting for age, BMI and renal disease the odds ratio (OR) was 6.88 (95% CI: 1.75-27.05; p = 0.006), suggesting about seven fold increase in the odds of preeclampsia development among those with long IBI compared to those with short IBI. Among women with the history of previous preeclampsia, association between IBI and preeclampsia status was not statistically significant (OR = 0.66; 95% CI: 0.07-4.99; p = 0.638) (Table 3).

**Table 3:** Interaction between interbirth interval and the history of previous preeclampsia

**History of previous preeclampsia**	**IBI**	**Unadjusted OR (95% CI)**	**Interaction term (95% CI)**	Adjusted OR^* ^**(95% CI)**	Adjusted Interaction term^* ^**(95% CI)**
Yes	Short(<5year)	1.00		1.00	
	Long(≥5year)	1.13 (0.17-7.24)	0.11	0.60 (0.07-4.99)	0.09
No	Short(<5year)	1.00	(0.01-1.01)	1.00	(0.01-0.96)
	Long(≥5year)	10.1 (3.12-32.73)		6.88 (1.75-27.05)	

BMI, Body Mass Index; IBI, Interbirth Interval.

*Adjusted for age, BMI, renal disease.

Table 4 presents the unadjusted and adjusted ORs for variables included in the final fitted model for all multiparous women who planned their pregnancies. The best fitting model included the IBI with a cut-point of 5 years, TTP, BMI, barrier methods of contraception and household monthly income. The Hosmer-Lemeshow test statistics was 5.0 (df = 8, Prob > chi2 = 0.76) indicating good calibration.

**Table 4:** Final fitted model for preeclampsia in multiparous women who had planned their pregnancies

**Variable**	**Univariate analysis**		**Multivariate analysis**	
	**Unadjusted OR**	**95% CI**	Adjusted OR^*^	**95% CI**
IBI (years)				
<5	1.00		1.00	
≥5	5.26	2.39-11.57	4.49	1.12-17.99
BMI (kg/m2)	1.19	1.11-1.28	1.20	1.04-1.38
Time to pregnancy				
<12 months	1.00		1.00	
≥12 months	5.40	1.78-16.38	5.99	1.39-25.83
Method of contraception				
Non barrier	1.00		1.00	
Barrier	1.70	0.81-3.56	3.63	0.90-14.67
Household monthly				
expenditure (AMD)				
≤100,000	1.00		1.00	
>100,000	5.26	2.39-11.57	4.49	1.12-17.99

AMD, Armenian Dram; BMI, Body Mass Index; IBI, Interbirth Interval.

*Adjusted for all variables in the table.

### IBI as a continuous variable

The findings remained consistent when IBI was treated as a continuous variable. After adjusting for age, BMI and renal disease, the odds ratio of having preeclampsia associated with one year increase in IBI was 1.19 (95% CI: 1.04-1.37; p = 0.012). The interaction term between IBI as a continuous variable and the history of previous preeclampsia was 0.77 (95% CI: 0.58-1.01; p = 0.059). Among women without history of previous preeclampsia after adjusting for age, BMI and renal disease, the OR was 1.32 (95% CI: 1.09-1.59; p < 0.0005), suggesting 32% increase in the odds of preeclampsia development with every year increase in IBI among women without history of previous preeclampsia. Among women with the history of previous preeclampsia, association between IBI as a continuous variable and preeclampsia status was not statistically significant (OR = 1.02; 95% CI: 0.80-1.30; p = 0.869).

## Discussion

This was the first epidemiologic study in Armenia investigating risk factors for preeclampsia development and associations of IBI and preeclampsia status. The results showed that IBI was statistically significantly associated with preeclampsia status after controlling for confounders among women without history of previous preeclampsia. Although our data confirmed that the risk of preeclampsia falls sharply after the first birth, the risk increases over time. Moreover, IBI of five years and more was associated with seven fold higher odds of preeclampsia development among women without a history of preeclampsia.

The association of longer than 12 months of TTP and preeclampsia status was consistent with the results of the study conducted by the Danish National Birth Cohort ongoing project among 45,610 women from 1998 to 2001 [22]. This study used TTP for the first time as a marker of fecundity for the association with preeclampsia status. The present case–control study identified TTP with cut-point of 12 months being a confounder for the relationship of IBI and preeclampsia status, suggesting that longer TTP might explain part of the increased odds of preeclampsia associated with long IBI. However, IBI was not adjusted for TTP in this study as only those women who planned their pregnancies reported TTP (small sample size). Future studies should assess the extent to which the longer TTP might account for the increased odds associated with long IBI.

The findings regarding increased odds of preeclampsia associated with increase in IBI were consistent with other studies that investigated the impact of interbirth interval on preeclampsia odds without stratifying women to history of previous preeclampsia [15,17-19]. An important finding of this study was detecting a statistically significant interaction between IBI and the history of previous preeclampsia. All other potential interaction terms that were investigated were not significant. For women without a history of previous preeclampsia, the odds of preeclampsia increased in subsequent pregnancy with increasing time between births whereas for women with history of previous preeclampsia the odds tended to decrease with increasing interval between births.

The findings regarding variables included in the final model as risk factors for preeclampsia development were consistent with the literature. The identified factors associated with preeclampeisa development were IBI [15,17-19], barrier methods of contraception [20], TTP with a cut-point of 12 months [22], BMI [8,9,15], and household monthly income [14].

The information regarding all variables included in our final model is easily measured and does not require any laboratory tests. Our study failed to investigate the association of preeclampsia status and diabetes, gestational diabetes, smoking, marital status, multiple pregnancy and partner change because of limited number of women with these characteristics in the sample. After excluding women with these characteristics from the analysis the results did not change.

The study had to rely on hospital records for the diagnosis of preeclampsia which is one of the limitations of the study. Problems with the diagnosis of preeclampsia involve great observer variability in measuring blood pressure and the commonly used dipstick analysis of a random urine sample rather than 24-hour urine analysis [28]. The process of measuring the exposure was not independent from the case–control status: the interviewer was aware of the women’s case or control status which could potentially lead to an interviewer bias.

All medical records were reviewed and uncertain diagnoses made based on using other than NHLBI criteria were excluded from the study to reduce the number of false positive diagnoses. Controls were selected by simple random selection. The same data sources were used to identify both cases and controls which increased the confidence that the cases and controls were coming from the same base population and the groups were comparable. Incidence-density approach of selecting controls is one of the strengths of the study. “The advantage for such an incidence-density selection strategy of controls is that it establishes comparability between cases and controls as to follow-up time for the detection of disease” [29]. Despite the relatively small number of participants, the actual power calculation demonstrated that the current sample size provided very high power to detect the true difference between the groups.

## Conclusion

The findings of this study showed that each additional year increase in IBI appeared to be a strong risk factor for preeclampsia development among women without history of previous preeclampsia. Further investigation of the role of the long IBI among women with history of previous preeclampsia may contribute to a new approach in understanding the etiology of preeclampsia and may be useful for developing further recommendations for this particular subgroup of women that are at higher risk for preeclampsia development in subsequent pregnancies.

## Competing interests

The authors declare that they have no competing interests.

## Authors’ contributions

AH contributed to the study design, coordinated the data collection, performed data analysis and conceptualized the scope of this paper and drafted the manuscript. HA and VP were responsible for the study concept and design, accuracy and completeness of data analysis, interpretation of the results and revising the manuscript. All the authors provided approval to the final version of the manuscript.

## Pre-publication history

The pre-publication history for this paper can be accessed here:

http://www.biomedcentral.com/1471-2393/13/244/prepub

## Supplementary Material

Additional file 1 Questionnaire. Click here for file
