# Supplementary material for: Interbirth interval and history of previous preeclampsia: a case–control study among multiparous women
Source: BMC Pregnancy Childbirth. 2013 Dec 27;13:244. doi: 10.1186/1471-2393-13-244 (PMC3877952; doi:10.1186/1471-2393-13-244)
Supplement: Additional file 1 — Questionnaire. [file 1471-2393-13-244-S1.pdf]

QUESTIONNAIRE

Date of the interview: **_ _ _ _ /_ _ _ _ /_ _ _ _** (Day) (Month) (Year)

Status: 1. Case
0. Control

1. **Maternity Home________**
2. **ID____________**
3. **3.Date of birth** _ _ _ _/_ _ _ _/_ _ _ _
   (Day) (Month)(Year)

4. Indicate the highest level of education that you have completed.

1. School (less than 10 years)
2. School (10 years)
3. Professional technical education (10-13 years)
4. Institute/University
5. Postgraduate

5. Have you been told by a physician that you had or have each condition below? *(Check **all** that apply)*

Disease	Yes	No
a. Diabetes		
b. Gestational Diabetes		
c. Renal (kidney) disease		

6. What was your birth weight? _________g 88. Don't know

7. What is your blood group, ___________ 88. Don't know
And RH ____________ 88. Don't know

Now I am going to ask questions about the pregnancy that ended ……_ _ __/ _ __/_ _ _
(Day) (Month)(Year)

8. What was your marital status during that pregnancy?

1. single
2. married
3. widowed
4. divorced
5. Refused to respond

9. How much did you weight before that pregnancy? _____________kg 88. Don't know

10. How tall are you? ____________cm 88. Don't know

11. What was the total number of people (including yourself and children under 18) living in your household during that pregnancy?_______

12. How many members of your household (including yourself) were employed during that pregnancy? _____

13. How would you rate your family's general standard of living during that pregnancy?

1. Substantially below average
2. Little below average
3. Average
4. Little above average
5. Substantially above average

88. Not sure/difficult to answer

14. During that pregnancy, the approximate amount of household income spent by all of your household members per month was

1. Less than 25,000 drams
2. From 25,000 – 50,000 drams
3. From 51,000 – 100,000 drams
4. From 101,000 – 250,000 drams
5. Above 250,000drams

99. Don't know

15. Please tell me whether your household or any member of it had the following working items during that pregnancy

Item	Yes	No
a. Individual heating system (Baxi)		
b. DVD player		
c. Automobile		
d. Automatic washing machine		
e. Personal computer		
f. Satellite		
g. Cell phone		
h. Vacation home/villa		

17. Were you employed during that pregnancy?

1. Yes
2. No ***(Go to the Q.21)***
3. I was a student

18. What were your working hours?

1. <4 hours
2. 4-8 hours
3. >8 hours
4. Other a. *specify* _________________

19. How had your work (studying) situation changed during pregnancy?
1. It did not change ***(Go to the Q.21)***
2. I had stopped working (studying)
3. I had reduced my working (studying) hours
4. Other ______a. *specify*

20. In which week of your pregnancy had your work (studying) situation changed?
_____________weeks of pregnancy

21. How many babies were born during that birth? ________

22. What was that baby's gender, weight, height at birth?
a.___ kg 88. Don't know b. ___ cm 88. Don't know c. gender 1.boy 2. girl 88. Don't know

a.___ kg 88. Don't know b. ___ cm 88. Don't know c. gender 1.boy 2. girl 88. Don't know

23. Have you/your partner at any time during one year before that pregnancy used the following methods to avoid becoming pregnant?
1. Condom
2. Diaphragm
3. IUD
4. Hormone injection
5. Oral contraceptives
6. Spermicides (foam, suppositories, cream)
7. Safe period
8. Withdrawal
9. Abstinence
10. Lactation Amenorrhea Method (breast feeding)
11. No such methods
12. Other (specify)____________________________

23. Was this pregnancy planned?
1. Planned
2. Partly planned
3. Not planned (***(Go to the Q.25)***
4. Don't know
5. Do not wish to answer

24. How long did you try to become pregnant before you succeeded?
1. became pregnant right away
2.1–2 months
3.3–5 months
4.6–12 months
5. More than 12 months
88. Don't know
99. Do not wish to answer

25. Did you take folic acid (vitamin B6) before/during that pregnancy?
1. Yes ___weeks before to _____ weeks of that pregnancy
2. Yes from____ weeks to ____weeks of that pregnancy
3. No
88. Don't know

26. Did you receive treatment from a doctor, nurse, or other health care worker to help you get pregnant with your new baby? (This may include infertility treatments such as fertility-enhancing drugs or assisted reproductive technology.)

1. Yes
2. No ***(Go to the Q.28)***

27. Did you use any of the following treatments *during the month you got pregnant* with your new baby? Check <u>all</u> that apply

1. Fertility-enhancing drugs prescribed by a doctor
2. Artificial insemination or intrauterine insemination
3. Other medical treatment a. specify___________________

28. Had the midwife or doctor told you that you have or have had high blood pressure during that pregnancy?
1. Yes a. specify the highest reading during that pregnancy_______/______99.Don't know
b. specify the time of onset ____weeks or ____month 99.Don't know
2. No
99. Don't know

29. Have you had high blood pressure without being pregnant?
1. Yes a. specify the highest reading before this pregnancy ________/______99.Don'tknow
2. No
99. Don't know

30. Have you ever smoked cigarettes?
1. Yes
2. No (***Go to Q.33)***

31. Did you smoke during that pregnancy? (*Go to Q.33*)
1. Sometimes _______ cigarettes per week ***(Go to Q.33)***
2. Daily_____________ cigarettes per day ***(Go to Q.33)***
3. I stopped smoking at ___ weeks of that pregnancy ***(Go to Q.33)***
4. No

32. When did you stop smoking?
1. _______months before that pregnancy
2. I did not smoke before that pregnancy

33. How many cigarette smokers, not including yourself, were living in your home during that pregnancy? ____

34. During that pregnancy, about how many hours a day, on average, were you in the same room with another person who was smoking? _____hours

35. Had anybody from your relatives (mother, sister, aunt etc.) have preeclampsia (high blood pressure) during pregnancy?
1. Yes a. specify______
2. No
88. Don't know

36. Have you been pregnant before that pregnancy? (Include all pregnancies that ended in life births, spontaneous or induced abortions, ectopic pregnancy and stillbirth as well)
1. Yes
2. No (finish)

37. Indicate all earlier pregnancies started from the first one, including all pregnancies that ended in life births, spontaneous or induced abortions, ectopic pregnancies and stillbirths as well. State the date/year the pregnancy ended and the gestational weeks of their termination. State whether or not you had preeclampsia (high BP) during that pregnancy.

N	Date/year of outcome	Life birth	Spontaneous abor-tion (<22 weeks)	Induced abortion (<22 weeks)	Ectopic pregnancy	Stillbirths (>22 weeks)	Gestational weeks at termi-nation	Preeclampsia (High BP during pregnancy)
1								
2								
3								
4								
5								
6								
7								

38. Were all your mentioned pregnancies from the same man?

1. Yes (Finish)
2. No
3. Refuse to respond

39. Have you had other pregnancy from the father of the baby born in _ __/ _ __/_ _ ___ .
(Day) (Month)(Year)
1. Yes
2. No
3. Refuse to respond

Thank you!
